# Frequency and voltage response of a wind-driven fluttering triboelectric nanogenerator

**DOI:** 10.1038/s41598-019-42128-7

**Published:** 2019-04-03

**Authors:** Martin Olsen, Renyun Zhang, Jonas Örtegren, Henrik Andersson, Ya Yang, Håkan Olin

**Affiliations:** 1 Department of Natural Sciences, Mid Sweden University, 851 70 Sundsvall, Sweden; 2 Department of Electronics Design, Mid Sweden University, 851 70 Sundsvall, Sweden; 3 CAS Center for Excellence in Nanoscience, Beijing Institute of Nanoenergy and Nanosystems, Chinese Academy of Sciences, Beijing, 100083 P. R. China

## Abstract

Triboelectric nanogenerators (TENG:s) are used as efficient energy transducers in energy harvesting converting mechanical energy into electrical energy. Wind is an abundant source of mechanical energy but how should a good triboelectric wind harvester be designed? We have built and studied a TENG driven by air flow in a table-top sized wind tunnel. Our TENG constitutes of a plastic film of size 10 cm × 2 cm which is fluttering between two copper electrodes generating enough power to light up a battery of LED:s. We measured the voltage and frequency of fluttering at different wind speeds from zero up to 8 m/s for three electrode distances 6 mm, 10 mm and 14 mm. We found that the frequency increases linearly with the wind speed with a cutoff at some low speed. Power was generated already at 1.6 m/s. We seem to be able to explain the observed frequency dependence on wind speed by assuming excitation of the film into different harmonics in response to von Kármán vortices. We also find that the voltage increase linearly with frequency. We anticipate that TENG:s of this design could be useful both as generators and speed sensors because they work at low air speeds.

## Introduction

**Figure 1:**
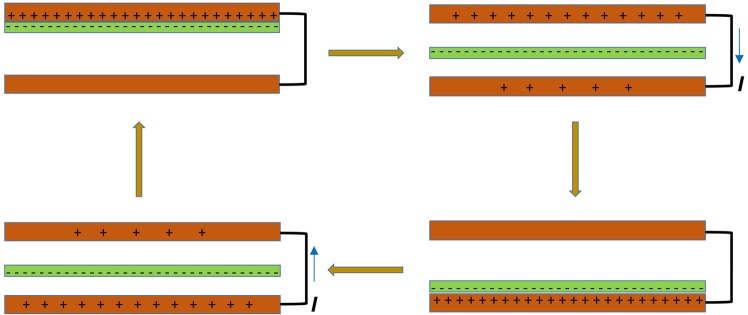
Drawing of the working principle of our triboelectric nanogenerator. The plastic FEP film (green) makes alternating contacts with the two electrodes (red) which generates an alternating voltage and current.

Triboelectric nanogenerators (TENG:s) are used as efficient energy transducers in energy harvesting to replace the need for batteries for small electronic devices^1–4^, or as sensors for different physical properties. For many types of generators containing non-metals the triboelectric charging seems to be done by ion transfer^5,6^ or by electron transfer between potential wells of atoms^7^ or by both mechanisms. At separation of the surfaces of the generator the so created charge differences induce an electric voltage that contains energy to be harvested. The separation can be done by linear sliding motion^8,9^, by rotary-sliding motion^10^ or by perpendicular surface separation^11,12^. A more evolved design with one dielectric film fluttering between two electrodes of the same metals are tested in this paper. The principle is shown in Fig. 1.

In this paper we have studied a triboelectric nanogenerator driven by air flow. How should such a nanogenerator be designed and what will be its characteristics? Many designs of a wind generator are possible: The most straightforward idea is to use an ordinary wind turbine and just replace the electromagnetic generator with a triboelectric generator^1^. Some more elaborate design is given by Yong *et al*.: They are considered a dielectric ball rolling inside a vortex chamber with one tangential air inlet and a central air outlet generating triboelectricity as the ball make contacts with the walls^13^. However, a triboelectric generator harvester can also be designed to include the turbine function in the generator part itself, allowing a considerable simpler design. For example Bae *et al*. studied a triboelectric generator in the form of a flag fluttering in the air stream close to an electrode^14^. Wang *et al*.^15^ made a wind-driven triboelectric nanogenerator device constituting of a two-sided clamped Kapton film that was fluttering between two copper electrodes. They measured the frequency and voltage as a function of length, width and thickness of the fluttering film and as a function of the electrode distance, with emphasis on the nanogenerator part.

**Figure 2:**
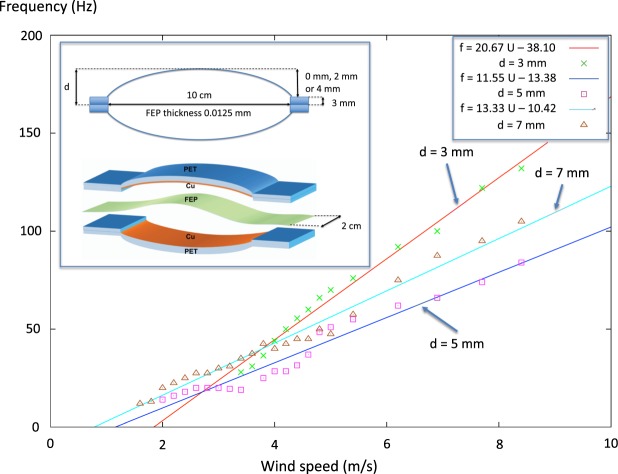
Fluttering frequency *f* as a function of wind speed *U* in the table-top sized wind tunnel (Omega mini wind tunnel, model WTM-1000) for three different half electrode distances *d* = 3 mm, 5 mm and 7 mm. The wind speed was set by adjusting a variable resistor and the speed was measured with an air speed meter belonging to the wind tunnel. Inset: Drawing of the TENG placed in the wind tunnel. The FEP plastic film is attached at both ends between two 3 mm thick plastic bricks. When the wind blows the FEP film is fluttering between the two copper electrodes placed at *d* = 3 mm away (parallel electrodes), *d* = 5 mm away (electrodes slightly bent outward) or *d* = 7 mm away (electrodes more bent outward) respectively.

Here, we report on a study on a wind nanogenerator similar to that of Wang *et al*., however, extended to also include a detailed study of the fluid dynamics using a table-top sized wind tunnel, allowing measurements of the frequency and voltage response as a function of wind speed. Our nanogenerator device, even simpler than the one reported by Wang *et al*., constituting of a two-sided clamped fluorinated ethylene-propylene (FEP) plastic ribbon placed between two parallel or two outwardly bent copper electrodes. At the start of the experiments the FEP plastic ribbon is mildly stretched and then clamped at the ends 10 cm apart, see inset in Fig. 2. However, because the clamping is not perfect and because the ribbon is elastic it can move a little so after a while the ribbon becomes relaxed and so becomes about 1 cm longer (i.e. 11 cm) between the clamps so that it can touch the electrodes when fluttering. This state is then stable generating a stable voltage signal for the two hours that we tested.

**Figure 3:**
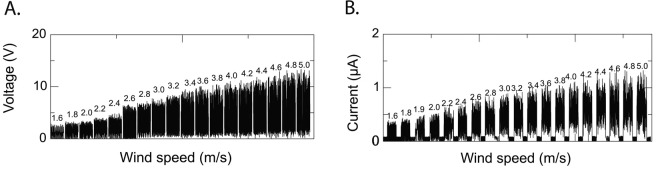
As the FEP plastic ribbon flutters between the electrodes an alternating voltage and an alternating current are obtained oscillating between their positive and negative maximum values. The raw signal is after that fed into a bridge rectifier (GJB2508 from Diodes Incorporated) making the negative values positive. The figures show the rectified time evolution of (**A**) the voltage at 18 different wind speeds and (**B**) the current at 19 different wind speeds. We see that the voltages and current for the larger wind speeds and thus higher fluttering and signal frequency are not going all the way down to zero. Assuming a small effective smoothing capacitance of the rectifier could explain this effect (the data sheet gives the typical value 85 pF). The effect of a smoothing capacitor is expected to be larger at higher frequencies and this agrees qualitatively with the data in (**A** and **B**).

**Figure 4:**
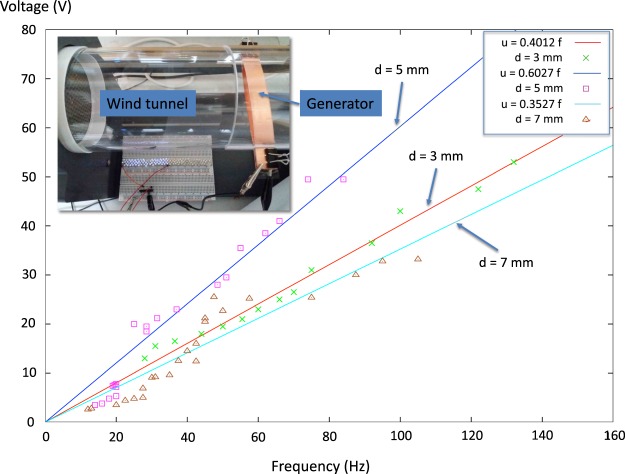
Voltage *u* as a function of vibration frequency *f* for three different half electrode distances *d* = 3 mm, 5 mm and 7 mm. The voltage is found to depend on the frequency *f* and the width *d*. We see that the voltage becomes larger for *d* = 5 mm than for *d* = 3 mm and *d* = 7 mm at the same frequency. Inset: Photography of the triboelectric nanogenerator mounted inside the table-top sized wind tunnel (Omega mini wind tunnel, model WTM-1000) at work, generating enough power to light up the LED:s on the circuit board.

The device was placed in a table-top sized wind tunnel (Omega mini wind tunnel, model WTM-1000) to let the air flow drive the FEP plastic film motion. The fluttering of the FEP plastic ribbon between the two copper electrodes generated alternating voltage and current with positive and negative maximum values. The raw signal was then fed into a bridge rectifier (GJB2508 from Diodes Incorporated) and the rectified signal data was recorded, see Fig. 3, giving frequency and voltage as a function of wind speed on which the plots in Figs 2 and 4 are based. The wind speed was measured with an air speed meter belonging to the wind tunnel.

We do in the paper a detailed study of the frequency and voltage response to wind speed using the three half electrode distances *d* = 3 mm, 5 mm and 7 mm (electrode distances 6 mm, 10 mm and 14 mm). We find that the frequency increases linearly with air speed with a cutoff at some low speed for each *d*. The generated power of our device is enough to power a battery of LED:s. In our device power started to be generated at wind speeds as low as 1.6 m/s. We also find that the voltage is proportional to the frequency at each *d*. We seems able to explain the main features of the data assuming the presence of von Kármán vortices exciting the plastic FEP ribbon into different harmonics of vibration. We anticipate that this design could be useful because it works at low wind speeds, suitable both as air speed meters and power generators.

## Analysis

First we look at the fluttering frequency dependence on the wind speed shown in Fig. 2 and try to explain it. Then we analyse the voltage dependence on the frequency shown in Fig. 4.

### Frequency versus wind speed

Flow induced vibration (FIV) includes many different mechanisms. For steady external flow we have two mechanisms: vortex induced (von Kármán) vibration (VIV) and fluidelastic vibration^16,17^. Vortex induced vibration is forced vibration due to fluid instabilities whereas fluidelestic vibration is due to flow disturbance coupled to a moving boundary guiding the fluid. In our case we seems to have both mechanisms at work: from the fixed electrodes there would be vortices generated but the fluttering film have moving boundaries and thus we have fluidelastic vibrations. The fluttering of a sheet, for example a flag or a two-sided clamped film, are in general a complex phenomenon where both the von Kármán vortices and the mass and elasticity of the sheet are affecting its fluttering behavior^18^. However, in our case the fluttering plastic FEP film is only 0.0125 mm thick and have thus very low mass. Using the size data in Fig. 2 and the density of FEP plastic 2150 kg/m^3^ we find the film mass to be 54 mg. If we thus assume it to be “massless” and that the tensile force in the film is zero because the way it is mounted in the generator, the only thing that is affecting the films fluttering would be the von Kármán vortices. When investigating oscillations in fluids the Strouhal number *St* = *fd*/*U* where *U* is a characteristic fluid speed and *d* is a characteristic distance perpendicular to the fluid flow is found to be of importance^19^. It is thus reasonable to assume that the frequency of fluttering of the plastic FEP film could be given by

1 $$f=St\frac{U}{d},$$

for some *St*. In our case we take the characteristic distance to be the triboelectric generator half width *d* in the air stream. The relation between the Strouhal number *St* and the Reynolds number *Re* = *Ud*/*ν*, where *ν* = 1.5 × 10^−5^ m^2^/s is the kinematic viscosity of air, can be deduced from the vorticity transport equation^20^:

2 $$St=A-\frac{B}{{Re}}.$$

The dimensionless constants *A* and *B* would be of the order unity. For a circular cylinder with diameter *d* as the obstacle generating the vortices, *A* = 0.21 and *B* = 5.4 was experimentally found. In our case we have not this geometry so the constants *A* and *B* would probably be different in our case^16,21^. Combining these two equations we obtain

3 $$f=\frac{A}{d}U-\frac{B\nu }{{d}^{2}}.$$

We see that the frequency increase linearly with *U* and has a cutoff at low air speeds given by *U*_*cut*_ = *Bν*/(*Ad*). Plotting *f* from Eq. (3) as a function of 1/*d* we obtain a parabola. Thus there exists an optimal *d* that maximizes the frequency *f* at constant *U*. Taking the derivative of Eq. (3) yields

4 $${d}_{opt}=\frac{2B\nu }{AU}.$$

Substituting this back into Eq. (3) we find the maximum frequency at a given air speed. We obtain

5 $${f}_{{\max }}=\frac{{A}^{2}{U}^{2}}{4B\nu }.$$

How does this model compare with our experimental results? If the model for the frequency Eq. (3) are correct the fit of the three curves in Fig. 2 for different *d* should all give the same values of the constants *A* and *B*. The data is showed in Table 1. We see that for *d* = 3 mm and 5 mm the constants are almost the same: *A* = 0.060 and *B* = 22. However, for *d* = 7 mm the constants *A* and *B* become larger: *A* = 0.093 and *B* = 34, indicating that the model is not entirely correct. The average values of the constants for the three curves are *A* = 0.071 and *B* = 26 (Note that if we would have taken the width of the generator 2*d* as the characteristic distance this would have doubled the *A*-constant and increased the *B*-constant four times). However, the ratio *A*/*B* is the same for all *d*, see Table 1. Thus *A* and *B* contains a common factor which is the same for the experiments with *d* = 3 mm and 5 mm but becomes 1.5 times larger for the experiment using *d* = 7 mm. Using our calculated constants *A* and *B* we can then write Eq. (3) for our three curves as

6 $$f=\xi \,(\frac{0.060}{d}U-\frac{22\nu }{{d}^{2}}),$$

where *ξ* = 1.0 for *d* = 3 mm and 5 mm and *ξ* = 1.5 for *d* = 7 mm.

**Table 1:** Calculated values of the constants *A* and *B* in Eq. (3) using the three fitted curves in Fig. 2.

Half width *d* (mm)	Calculated *A*	Calculated *B*	Ratio *A*/*B*	Harmonic *n*
3	0.062	23	0.0027	2
5	0.058	22	0.0026	2
7	0.093	34	0.0027	3
Average	0.071	26	0.0027	—

If the model Eq. (3) are correct all three fitted values of *A* and *B* respectively in the table should be the same. The three equations for the fitted curves are for the *d* = 3 mm case *f* = 20.67 × *U* − 38.10, for the *d* = 5 mm case we have *f* = 11.55 × *U* − 13.38 and for the *d* = 7 mm case we have *f* = 13.33 × *U* − 10.42. The constants *A* and *B* are not the same for all *d* but the ratios *A*/*B* are the same. To the rightmost in the table are the different vibrational harmonics showed suggested to explain the differences in *A* and *B*.

The physical mechanism behind the difference in *ξ* is unknown but a difference in the small tensile force *T* that could be present in the fluttering plastic film between the different experiments may possibly be an explanation. A well known model for the resonance frequency of a free non-driven vibrating string of length *L* and linear density *μ* with a tensile force *T* is given by

7 $$f=\frac{n}{2L}\sqrt{\frac{T}{\mu }},$$

where *n* = 1, 2, 3, … gives the different harmonics. An increase in *T* yields a higher frequency. However, the values of *ξ* = 2/2 and *ξ* = 3/2 are so “integer like” indicating the possibility that *ξ* = 1 and *ξ* = 1.5 are just corresponding to different harmonics at the same force *T*. If Eq. (7) can be used *ξ* = 1 could correspond to *n* = 2 and *ξ* = 1.5 could correspond to *n* = 3. We finally note in Fig. 2 that the *d* = 5 mm and *d* = 7 mm lines almost coincide forming one single line as we see from the fitted equations for these curves shown in the legend of Table 1.

### Voltage versus frequency

Analytical calculation of charge and voltage of a triboelectric generator in contact-mode can be found in Yang *et al*.^11^. The voltage is in this reference found to increase linearly with the frequency which our experiments confirm see Fig. 4. For *d* = 3 mm, 5 mm and 7 mm we found *u* = 0.401248 × *f*, *u* = 0.602743 × *f* and *u* = 0.352747 × *f* respectively. The fitted lines for *d* = 3 mm and *d* = 7 mm almost coincide. Given the frequency *f* the voltage thus becomes largest at *d* = 5 mm.

Why is there an optimum *d* giving the voltage *u* a maximum at constant frequency? We could give two reasons: (A): Approximating the fluttering film and electrode as a capacitor of capacitance *C* one would expect the voltage *u* to increase with the half width *d* as

8 $$u=\frac{Q}{C}=\frac{2Qd}{{\varepsilon }_{0}S},$$

where *Q* is the charge, *S* is the capacitor area, 2*d* is the distance between the “plates” and *ε*_0_ is the permittivity of air. (B): The impact speed *v* of the plastic ribbon fluttering between the two copper electrodes is approximately

9 $$v=\frac{2d}{P/2}=4df,$$

where *P* = 1/*f* is the period time for one oscillation. The charge transfer *Q* increases with contact force^22,23^. The contact force *F* in turn increases with the impact speed *v* because the impulse *I* = *Ft* = 2 *mv* where *t* is the contact time. This two reasons (A) and (B) suggest that the voltage should increase with the half width *d*. We see that as *d* increases from 3 mm to 5 mm the voltage increases in qualitatively agreement with this.

However, for the larger *d* = 7 mm the voltage decrease compared to the 5 mm case, see Fig. 4. This effect can be due to that for this *d* an higher harmonic *n* = 3 is excited instead of the lower harmonic *n* = 2 which may be excited at *d* = 3 mm and 5 mm. Different harmonics has different geometrical shapes affecting the contact area with the electrodes and thus can change the charge transfered and so the voltage.

## Discussion

We have investigated the frequency and voltage response of a wind driven triboelectric nanogenerator. Our proposed von Kármán model Eq. (3) for the frequency as a function of wind speed and nanogenerator width seemed at first sight not to be entirely correct. However, the fit of data to this model showed interesting features giving the model some merit: that the quota *A*/*B* is constant for all *d* means that the measured frequency is proportional to the von Kármán vortex shedding frequency for all widths. The proportionality factor *ξ* is also the same for two out of three widths. Our explanation of this is that the two different *ξ* found for the three widths simply correspond to two different excited vibration harmonics of the fluttering film.

The measured voltage dependency on the frequency and nanogenerator width show that there exists an optimal width making the voltage maximum at constant frequency. From a capacitor model Eq. (8) we seems to be able to explain this: We see from the model first that as the with *d* increases the voltage should increase at constant charge. The experiments confirm this as the half width increases from 3 mm to 5 mm. However, according to the experiment, increasing the width over the optimum to *d* = 7 mm will decrease the voltage. This latter effect can however be due to that we here have another excited vibrational harmonic in the film. Different harmonics give different shape of the film and thus different contact area with the electrodes affecting the triboelectric charging.

How does the impedance of the load affect the harvested power? Impedance matching of the load impedance yields the maximum power into the load. If we have a circuit with a load in series with the source we have the well known condition for maximum power: The resistance of the load should be the same as the resistance of the source and the reactance of the load (capacitive or inductive) should be equal in size and have opposite sign to the reactance of the source. This means that if the source is capacitive then the load should be inductive. The triboelectric generator is in Hu *et al*. modeled as a voltage source in series with a capacitor^24^.

We anticipate that a wind driven triboelectric nanogenerator of this design can be useful because it can generate power already at as low air speeds as 1.6 m/s making them suitable both as air speed sensors as well as power generators.
